# Epigenetic Regulation of Alternative Splicing: How LncRNAs Tailor the Message

**DOI:** 10.3390/ncrna7010021

**Published:** 2021-03-11

**Authors:** Giuseppina Pisignano, Michael Ladomery

**Affiliations:** 1 Department of Biology and Biochemistry, University of Bath, Claverton Down, Bath BA2 7AY, UK; 2 Faculty of Health and Applied Sciences, University of the West of England, Coldharbour Lane, Frenchay, Bristol BS16 1QY, UK

**Keywords:** long non-coding RNAs, alternative splicing, splicing factors, post-transcriptional regulation

## Abstract

Alternative splicing is a highly fine-tuned regulated process and one of the main drivers of proteomic diversity across eukaryotes. The vast majority of human multi-exon genes is alternatively spliced in a cell type- and tissue-specific manner, and defects in alternative splicing can dramatically alter RNA and protein functions and lead to disease. The eukaryotic genome is also intensively transcribed into long and short non-coding RNAs which account for up to 90% of the entire transcriptome. Over the years, lncRNAs have received considerable attention as important players in the regulation of cellular processes including alternative splicing. In this review, we focus on recent discoveries that show how lncRNAs contribute significantly to the regulation of alternative splicing and explore how they are able to shape the expression of a diverse set of splice isoforms through several mechanisms. With the increasing number of lncRNAs being discovered and characterized, the contribution of lncRNAs to the regulation of alternative splicing is likely to grow significantly.

## 1. Introduction

In the late 1970s, researchers were interested in gaining a better understanding of the mechanisms of adenoviral gene expression when they noticed something unusual, a long adenoviral transcript hybridized to the viral genome forming a three-stranded mRNA:DNA hybrid structure with an intervening DNA sequence that did not match the mature mRNA [1]. It then became apparent that these intervening sequences were in fact introns and that the primary transcript is made by a succession of exonic and intronic sequences. In what is now thought to be a mainly co-transcriptional process, introns are ‘spliced’ out from the mRNA precursor (pre-mRNA) and the exons joined together through two transesterification reactions catalysed by a complex molecular machinery consisting of five small nuclear ribonucleoproteins (snRNPs), called the spliceosome [2]. Over the past decades it has become clear that pre-mRNA splicing is a widespread phenomenon across eukaryotes and that a single gene can generate multiple transcripts often encoding different proteins by a process known as alternative splicing (AS) [3]. Many types of AS are possible, including “cassette exons”, “alternative 5′ and 3′ splice sites”, “alternative first exons” (through different promoters), “alternative last exons” (through different polyadenylation sites), “mutually exclusive exons” and “retained introns” [4]. Fundamental to the process of AS is the definition of the precise location of 5′ (donor) and 3′ (acceptor) splice sites and the assembly of the spliceosome complex. The first relies on splicing factors (SFs), a category of RNA-binding proteins (RBPs) expressed in a tissue and stage-specific way that recognize regulatory elements within exons and introns. Importantly, SF activity is in turn modified by splicing factor kinases and phosphatases activated through cell signaling mechanisms.

**Figure 1:**
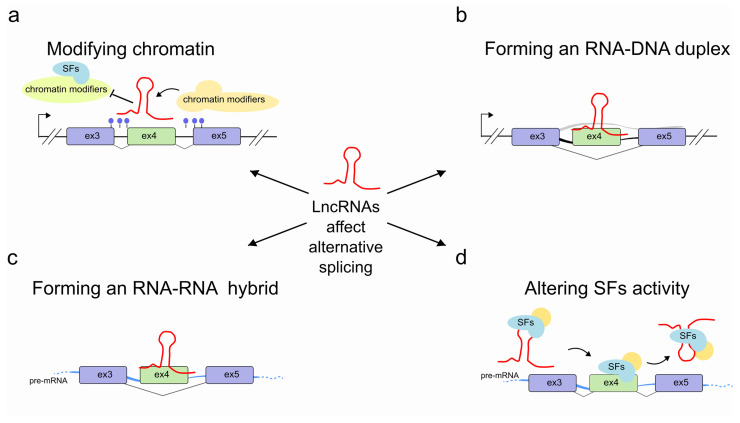
Regulation of pre-mRNA splicing by lncRNAs. LncRNAs (red) are able to control pre-mRNA splicing by (**a**) modifying chromatin accessibility through recruiting or impeding access to chromatin modifying complexes at the transcribed genomic locus. In some cases, this might result in more drastic long-range structural changes; (**b**) interacting with the transcribed genomic locus through an RNA-DNA hybrid; (**c**) hybridizing with the pre-mRNA molecule (light blue); (**d**) promoting SF recruitment or by sequestering SFs into specific subnuclear compartments, thereby interfering with SF activities.

AS greatly enhances proteome diversity and represents an essential aspect of gene expression in development, normal physiology and disease across eukaryotes [5], from single-celled yeast to humans [6]. The advent of high-throughput sequencing technologies revealed that ~92–94% of human multi-exon genes are alternatively spliced [7], increasing the interest in understanding the mechanisms underpinning its regulation. With the discovery and growing importance of non-coding RNAs, the nature of AS regulation has become more complex. Both short (<200 nt) and long (>200 nt) non-coding RNAs can contribute to the regulation of AS in many different ways; either indirectly by regulating the activity of splice factors; or directly, by interacting with pre-mRNAs. Long non-coding RNAs (lncRNAs) are particularly well suited to these roles due to their demonstrated capacity to act as regulatory molecules that modulate gene expression at every level. Either alone, or in association with partner proteins, these long RNA polymerase II transcripts have been shown to take part in a wide range of developmental processes and disease in complex organisms [8,9,10]. Here, we review the current knowledge of the multiple mechanisms through which lncRNAs contribute to the regulation of AS (Figure 1 and Table 1).

## 2. LncRNAs Regulate Alternative Splicing through Chromatin Modification

The eukaryotic genome is tightly packaged into chromatin fibers consisting of DNA wrapped around nucleosomes made of histone proteins. Post-translational modifications (PTMs), such as methylation, acetylation, phosphorylation, and ubiquitination, occur on the histone tails that are functionally linked to the epigenetic regulation of gene expression. By defining the accessibility to chromatin, histone modifications demarcate amenable or silenced chromatin domains which ultimately reflect the activity of gene transcription. An intimate relationship exists between lncRNAs and chromatin conformation [11,12]. LncRNAs regulate chromatin modifications by recruiting or directly interacting with histone-modifying complexes or enzymes at specific chromosomal loci; these in turn modulate gene transcription [13,14,15,16,17,18]. Histone modification signatures can also influence AS through a chromatin-reading protein which acts as an adaptor linker between the RNA polymerase II (RNAPII) and the pre-mRNA splicing machinery [19]. Several studies have also demonstrated that the local chromatin context influences the RNAPII elongation rate which in turn affects AS [20,21,22].

**Figure 2:**
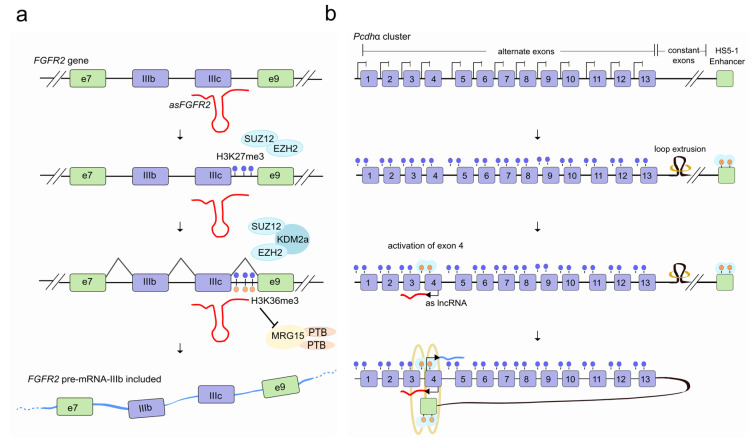
LncRNAs regulate alternative splicing through chromatin modification. (**a**) In epithelial cells, the antisense lncRNA *asFGFR2* (red), recruits the Polycomb-group proteins EZH2 and SUZ12 to the *FGFR2* gene locus and allows H3K27me3 deposition (blue lollipop) and a decrease in methylation of H3K36me3 (orange lollipop) by the recruitment of the H3K36 demethylase KDM2a. As a result, the chromatin-splicing adaptor complex MRG15–PTB1 can no longer bind to exon IIIb, which is then included in the *FGFR2* transcript (light blue). (**b**) The activation of a specific antisense lncRNA (as lncRNA; red) at the *Pcdh*α promoter of one (out of 13) alternate first exon promotes proximal DNA demethylation (orange lollipop) and CTCF (turquoise) recruitment and favors the interaction between the selected promoter and a distant HS5-1 enhancer by a long-range DNA loop. This ultimately triggers sense transcription (light blue) of the corresponding selected *Pcdh*α first-exon which is individually spliced to a downstream constant region to form a distinct transcript.

A possible lncRNA-mediated crosstalk between histone modifications and the pre-mRNA splicing machinery has also been proposed [23]. Cell type–specific splicing of the gene encoding the fibroblast growth factor receptor 2 (*FGFR2*) is now known to rely on the methylation state of the *FGFR2* locus. In mesenchymal stem cells, *FGFR2* is enriched in di- (me2) and tri-methylated (me3) histone H3K36, which inhibits the inclusion of the alternatively spliced exon IIIb. *FGFR2* is, in contrast, devoid of H3K36 methylation in epithelial cells. The cell-specific switch in splicing is made possible by an evolutionarily-conserved nuclear antisense lncRNA (*asFGFR2*), transcribed within the human *FGFR2* locus and exclusively expressed in epithelial cells. By recruiting Polycomb-group proteins and the histone lysine-specific demethylase 2a (KDM2a) to the locus, *asFGFR2* ensures the deposition of H3K27me3 and a decrease in H3K36me2/3. This impairs both the binding of the chromatin-binding protein MRG15 for H3K36me2/3 [24] and the recruitment, via protein-protein interactions, of the negative splicing regulator PTBP1 to exon IIIb [19]. Through this combined action, the chromatin-splicing adaptor complex MRG15–PTBP1 can no longer inhibit the inclusion of exon IIIb favoring the epithelial-specific AS of *FGFR2* [23] (Figure 2a).

Chromatin structure is itself likely to play an important role in modulating the effects of transcription on AS [25]. In particular, the tri-dimensional chromatin organizer CCCTC-binding factor (CTCF) has been shown to bind target DNA sites located within an alternative exon creating a roadblock to transcriptional elongation that favors exon inclusion into mature mRNA [26]. Several lncRNAs appear to control important aspects of chromatin organization including chromatin looping, either remaining tethered to the site of transcription or moving over distant loci [27,28]. Interestingly, lncRNAs can efficiently remove structural roadblocks in chromatin by CTCF eviction [29,30]. A fascinating lncRNA-mediated mechanism modulates the diversity of transcripts at the complex Protocadherin (*Pcdh*) α gene cluster [31]. Each *Pcdh*α gene of the cluster functions as a ‘variable’ first exon (out of 13) that is individually spliced to a downstream constant region to form distinct transcripts, differentially expressed in individual neurons and important for neuronal self-identity. The stochastic expression of 13 alternate exons is driven by their own promoter, each of which is equally likely to be activated by a long-range DNA loop interaction between a selected *Pcdh*α promoter and a downstream enhancer, called “hypersensitivity site 5-1” (HS5-1) [32,33,34,35]. The *Pcdh*α gene choice involves the selective activation of a specific antisense lncRNA located at the promoter of the first exon of each *Pcdh*α alternate gene. By promoting DNA demethylation, the antisense transcript recruits CTCF at sites proximal to the relative promoter and favors the promoter-enhancer interaction which ultimately triggers the sense transcription of the corresponding selected first exon [31] (Figure 2b). Further studies will be required to understand if other clustered genes share a similar mechanism. It will also be of interest to determine how frequently mechanisms involving lncRNAs, among the thousands transcribed, mediate chromatin structure changes that result in AS.

## 3. LncRNAs Regulate Pre-mRNA Splicing through RNA-DNA Interactions

LncRNAs can tether DNA forming an RNA-dsDNA triplex by targeting specific DNA sequences and inserting themselves as a third strand into the major groove of the DNA duplex [30,36]. These are known as R-loops; three-stranded nucleic acid structures, composed of RNA–DNA hybrids, frequently formed during transcription. Aberrant R-loops are generally associated with DNA damage, transcription elongation defects, hyper-recombination and genome instability [37].

**Figure 3:**
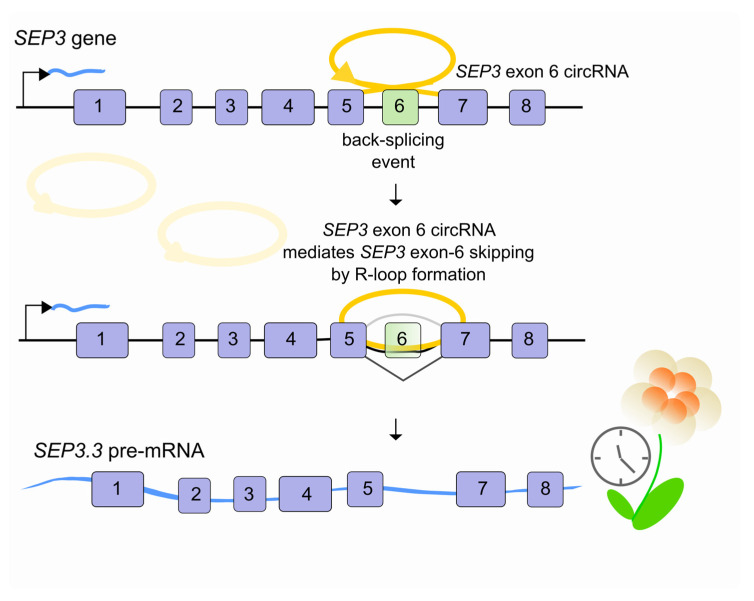
LncRNAs regulate pre-mRNA splicing through an RNA-DNA interaction. In *Arabidopsis thaliana*, when the *SEP3* gene is transcribed, exon 6 can be back-spliced into a circular RNA (*SEP3* exon 6 circRNA, yellow) which interacts directly with its parental genomic locus. By forming RNA–DNA hybrids (R-loops), *SEP3* exon 6 circRNA favors exon-6 skipping of its linear cognate and promotes the *SEP3.3* mRNA (light blue) isoform accumulation which in turn affects flowering time.

Recent lines of evidence indicate a potential role for R-loops in alternative pre-mRNA splicing. A class of lncRNAs, the so-called circular RNAs (circRNAs), have recently been characterized [38,39,40]. These abundant, conserved transcripts originate from a non-canonical AS process (back-splicing) leading to the formation of head-to-tail splice junctions, joined together to form circular transcripts. Recent studies suggest that they are clearly involved in multiple aspects of normal physiology, development and disease [41]. Since most circRNAs are derived from the middle exons of protein-coding genes [42], their biogenesis can itself affect splicing of their precursor transcripts and lead to altered gene expression outcomes [43]. For example, in *Arabidopsis thaliana*, the circular RNA derived from exon 6 of the *SEPALLATA3* (*SEP3*) gene increases the abundance of the cognate exon-skipped alternative splicing variant (SEP3.3 isoform) which in turn drives floral homeotic phenotypes [44]. This is made possible because *SEP3* exon 6 circRNA tethers to its cognate DNA locus through an R-loop promoting transcriptional pausing, which coincides with SF recruitment and AS [45,46,47] (Figure 3). Whether or not other lncRNAs are involved in similar processes in plants or other organisms remains to be investigated.

## 4. LncRNAs Regulate Pre-mRNA Splicing through RNA-RNA Interactions

Over the past decades, antisense transcripts have been characterized as being widespread throughout the genomes of the vast majority of organisms [48,49,50]. It is estimated that more than 30% of annotated human transcripts have at least one cognate antisense transcript [50]. Although generally low in abundance and over 10-fold less expressed than their counterpart sense transcripts [50], antisense RNAs have been widely implicated at almost all stages of gene expression, from transcription and translation to RNA degradation [51]. A considerable proportion of genes that express multiple spliced isoforms has been associated with antisense transcription, suggesting that antisense-mediated processes could be a common mechanism to regulate AS [52]. Therapeutic strategies based on antisense-mediated exon skipping and aimed at changing the levels of alternatively spliced isoforms or at disrupting open reading frames have been also developed [20]. For example, an antisense oligoribonucleotide (AON) approach efficiently restores the open reading frame of the *DMD* gene and generates functional dystrophin by inducing exon skipping [53].

**Figure 4:**
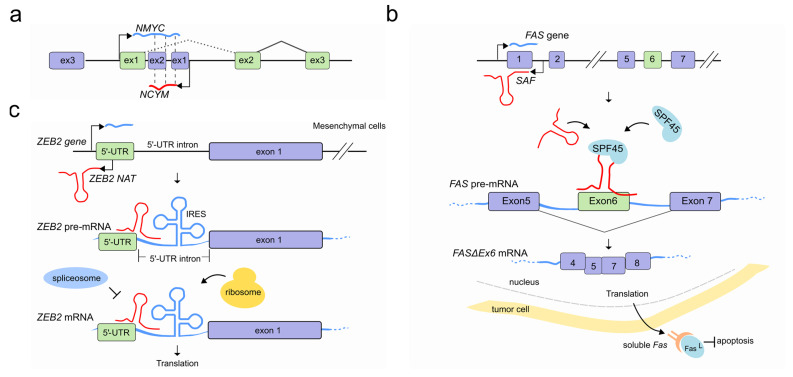
LncRNAs regulate pre-mRNA splicing through an RNA-RNA interaction. (**a**) NAT (red) at the *NCYM* gene modulates splicing of the *NMYC* mRNA (light blue) forming a sense-antisense RNA-RNA duplex which results in an intron-retained *NMYC* mRNA isoform population. (**b**) In tumor cells the natural antisense *SAF* (red) is transcribed from the first intron of *FAS* gene and interacts with both *FAS* pre-mRNA (light blue) at 5–6 and 6–7 exon junctions and the human SFP45 to facilitate the AS and exclusion of exon 6. The accumulation of the exon 6-skipped alternatively spliced variant of *FAS* pre-mRNA (*FASΔEx6* mRNA) leads to the production of a soluble Fas (sFas) protein that binds FasL and makes tumor cells resistant to FasL-induced apoptosis. (**c**) After EMT, Snail1 transcription factor induces the co-transcription of *ZEB2* NAT (red) in mesenchymal cells. *ZEB2* NAT hybridises with a region of the *ZEB2* pre-mRNA (light blue) encompassing the 5′ splice site of a 3 kb-long 5′-UTR intron. This RNA-RNA duplex prevents both the binding of the spliceosome and the subsequent removal of the 5′-UTR intron. The resulting mRNA contains the full isoform of the 5′-UTR, including an internal ribosome entry site (IRES) proximal to the *ZEB2* AUG, which favors translation. In absence of *ZEB2* NAT (epithelial cells) instead the removal of the 5′-UTR intron results in an mRNA containing a sequence that inhibits scanning by the ribosomes and therefore prevents translation of ZEB2 protein (not shown).

Identified in multiple eukaryotes, Natural Antisense Transcripts (NATs) are a class of long non-coding RNA molecules, transcribed from both coding and non-coding genes on the opposite strand of protein-coding ones [54]. Regardless of their genomic origin, NATs can hybridize with pre-mRNAs and form RNA-RNA duplexes. In some cases, a double function is also possible, and NATs can encode for proteins on one hand, while at the same time working as non-coding molecules modulating the splicing of a neighbouring gene’s transcript [55]. At the oncogene *NMYC* locus, for example, the cis-antisense gene *NCYM* located at the first *NMYC* intron has recently been shown to encode a protein that regulates the genesis and progression of human neuroblastomas that is associated with unfavorable prognosis [56]. However, previous studies have classified the corresponding transcript as a NAT able to modulate, via sense/antisense RNA-RNA duplexes, the processing of *NMYC* pre-mRNA resulting in a population of *NMYC* mRNA splice isoforms that retain the first intron [57] (Figure 4a).

Overlapping antisense transcription has been shown to modulate AS at the thyroid hormone receptor alpha (*THRA*) locus [58]. This locus encodes two overlapping mRNAs, *α1* and *α2* corresponding to TR-α1 and its splice variant TR-α2, which differ at the 3′-end because of the presence of a third overlapping mRNA, *NR1D1* (also known as *Rev-erbAα).* The latter is transcribed in the opposite direction at the 3′-end of *α2*, but not *α1* mRNA. It has been suggested that the relative abundance of the *NR1D1* RNA prevents the splicing of *α2,* likely through RNA-RNA base pairing, thereby favoring the formation of the non-overlapping *α1*. Consistent with this hypothesis, other studies noted a positive correlation between the *α2/α1* isoform ratio and the level of *NR1D1* mRNA in cells [58,59]. Therefore, relatively modest changes in splice site selection of *α1* and *α2* caused by naturally occurring antisense RNAs might cause major changes in cellular thyroid hormone-responsiveness with a broader physiological impact.

NATs that drive AS during programmed cell death (apoptosis) have also been reported. The FAS gene encodes for a receptor protein which usually binds its Fas ligand (FasL) and triggers the apoptotic process. At the *FAS* locus, the lncRNA SAF is transcribed in reverse orientation and from the opposite strand of the first intron of FAS. In tumor cells, *SAF* transcription promotes the formation of the exon 6-skipped spliced variant of *FAS* pre-mRNA (*FASΔEx6*) by interacting with both the *FAS* pre-mRNA, predominantly at exon 5/6 and exon 6/7 junctions, and the human splicing factor 45 (SPF45). The resulting splicing variant lacks the transmembrane domain which gives more solubility to the isoform (sFas) and protects tumor cells against FasL-induced apoptosis [60] (Figure 4b).

Reverse transcription can affect pre-mRNA splicing by masking specific splice sites and preventing their processing. A remarkable example of how NATs can affect the splicing and in turn increase mRNA translation efficiency is the human *ZEB2* gene (zinc-finger E-box-binding homeobox 2). Boosting the translation of ZEB2 repressor is one of the ways by which E-cadherin repression is initiated by the transcriptional factor Snail1 during epithelial-mesenchymal transition (EMT). Normally, the *ZEB2* 5′-UTR contains a structural intronic motif that works as an internal ribosome entry site (IRES) which is spliced out to hinder ZEB2 translation. However, once EMT is triggered, Snail1 induces the transcription of a *ZEB2* NAT which is transcribed from the opposite strand of the *ZEB2* locus, covering the 5′ splice site of the *ZEB2* 5′-UTR. *ZEB2* NAT prevents the recognition of the splice sites by the spliceosome by RNA-RNA duplex interaction with *ZEB2* mRNA and promotes the subsequent inclusion of the intron present in the *ZEB2* 5′ UTR, thereby promoting ZEB2 translation [61] (Figure 4c).

Masking canonical splicing sites has also been linked with the most common form of dementia, Alzheimer’s disease (AD). Sortilin-related receptor 1 (SORL1) expression is generally reduced in brain tissues from individuals with AD [62] suggesting a potential role in AD pathogenesis [63,64]. The importance of this receptor is underlined by the recent demonstration that SORL1 downregulation promotes amyloid precursor protein (APP) secretion and subsequently an increase of neurotoxic β-amyloid peptide (Aβ) [65,66]. A 300 nt antisense non-coding RNA transcribed by RNA polymerase III, called *51A*, maps to the intron 1 of the *SORL1* gene and, by pairing with the *SORL1* pre-mRNA, drives a splicing shift of SORL1 from the canonical full-length protein variant A to an alternatively spliced shorter protein form (variant B). This process results in the decreased synthesis of SORL1 variant A and is associated with impairing processing of APP, leading to increase of Aβ formation [67].

Antisense transcripts that cause a shift in isoform balance occur also at the *GPR51* locus, hosting the antisense lncRNA *17A* on its intron 3. LncRNA *17A* expression is induced by inflammatory molecules and leads to the production of the GABAB R2 protein isoform devoid of transduction activity and the concomitant down-regulation of the canonical full-length GABAB R2 variant, which impairs GABAB signaling. The change in the ratio of the two isoforms was found to be linked to AD. Increased levels of *17 A* expression have been found in patient brains, suggesting a role of this lncRNA in *GPR51* splicing regulation to preserve cerebral function [68].

Alternative isoform expression can also be controlled by antisense transcription via transcription attenuation (transcription RNAPII pausing and/or premature termination). A recent study shows that during specific differentiation stages in mouse embryonic stem cells (mESCs), the expression of two novel antisense enhancer-associated RNAs, *Zmynd8as* and *Brd1as*, is associated with shorter overlapping sense transcript isoforms with alternative termination sites [69], a phenomenon similarly found affecting the length of sense mRNAs of genes in a single operon in some bacteria [70]. Whereas the mechanism through which isoform specificity is achieved via enhancer-associated antisense RNAs has not been totally elucidated, this example enhances the corollary of antisense-mediated splicing mechanisms. A similar transcription attenuation mechanism mediating splicing is likely to occur at other genomic loci occupied by overlapping coding and non-coding genes [52].

## 5. LncRNAs Regulate Pre-mRNA Splicing by Modulating the Activity of Splicing Factors

As well as modifying AS by altering the chromatin landscape, through transcription, or through direct nucleic acid interactions, lncRNAs also interact in a dynamic network with many SFs and their pre-mRNA target sequences to modulate transcriptome reprogramming in eukaryotes. 

LncRNAs that are notoriously associated with pre-mRNA splicing are the nuclear *MALAT1/NEAT2* and *NEAT1,* both known to regulate the localization and phosphorylation status of SFs, and differentially expressed in a wide range of tissues in human and mouse. They are localized to specific subnuclear domains mainly in the nuclear speckle periphery, also known as paraspeckles (*NEAT1*); while *MALAT1/NEAT2* is part of the polyadenylated component of nuclear speckles [71].

**Figure 5:**
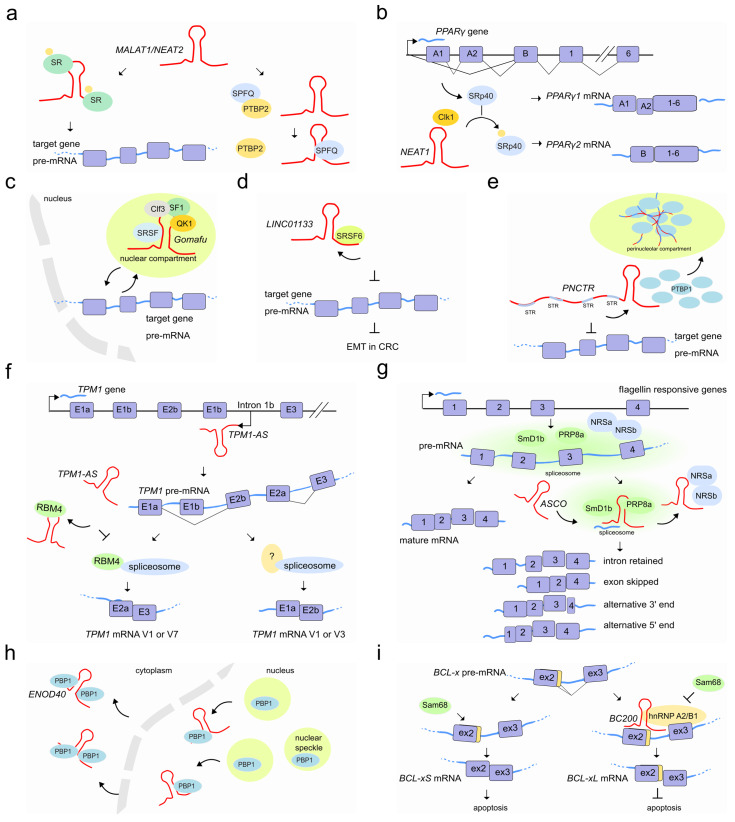
LncRNAs regulate pre-mRNA splicing by recruiting or sequestering splicing factors into subnuclear compartments. (**a**) Left, *MALAT1/ NEAT2* (red) is responsible of phosphorylated/dephosphorylated SFs shuttle from nuclear speckles to target mRNAs and cytoplasm. Right, *MALAT1/ NEAT2* in colon cancer. The binding of SFPQ with *MALAT1/ NEAT2* causes the disruption of the splicing regulator complex SFPQ-PTBP2 and the release of PTBP2. (**b**) During adipogenesis, the lncRNA *NEAT1* (red) interacts with the CLK1 splicing factor kinase (orange) and regulates *PPARγ* gene splicing by modulating SRp40 (light blue, also known as SRSF5) phosphorylation status (light orange). When Srp40 is phosphorylated, the *PPARγ* pre-mRNA is mainly processed into the *PPARy2* mRNA, whereas when dephosphoryled, Srp40 promotes the accumulation of the PPARy1 isoform. (**c**) *Gomafu* (red) sequesters multiple splicing factors (e.g., QKI, SRSF1, SF1, Clf3) in nuclear compartments and after specific stimuli/conditions it releases them in the nucleus to then direct the alternative splicing of pre-mRNA target genes (light blue) such as the schizophrenia-associated genes. (**d**) The lncRNA *LINC01133* (red), by sequestering the splicing factor SRSF6, impairs the alternative splicing events on target pre-mRNA genes which ultimately lead to the inhibition of EMT and metastasis in colorectal cancer (CRC). (**e**) *PNCTR* (red), contains hundreds of short tandem repeats (STR) to bind and sequester a substantial fraction of PTBP1 in the perinucleolar compartment. (**f**) Sense and antisense *TPM1* gene cotrascription results in both *TPM1* pre-mRNA (light blue) and lncRNA *TPM1-AS* (red). The latter is then able to sequester RBM4 protein, forcing the splicing of *TPM1* pre-mRNA (likely in cooperation with other protein partners) toward RBM4-deprived specific isoforms (V1 or V3). (**g**) LncRNA *ASCO* (red) associates with the two core components of the spliceosome SmD1b and PRP8a (green) and concomitantly sequesters NSRa and b proteins (light blue). By this mechanism *ASCO* enhances transcriptome diversity in response to flagellin, resulting in a variety pool of isoforms. (**h**) *ENOD40* is recognized by MtRBP1 (here RBP1 for simplicity) and is responsible of its nucleocytoplasmic trafficking and accumulation into cytoplasmic granules, likely modulating RBP1-dependent splicing. (**i**) Left, *BCL*-x pre-mRNA interacts with Sam68 that promotes pre-mRNA splicing in the apoptotic isoform *BCL-xS*. Right, the presence of *BC200* lncRNA and the recruitment of the hnRNP A2/B1 splicing factor interferes with the association of Sam68 and promote *BCL-x* splicing into the anti-apoptotic *BCL-xL*.

*MALAT1/NEAT2* regulates splicing by modulating the activity of the conserved family of serine/arginine (SR) splicing factors by modifying their localization and phosphorylation [72] through shuttling between speckles and the sites of transcription, where splicing occurs [73]. In human cells, *MALAT1/NEAT2* knockdown enhances the phosphorylated pool of SR proteins, displaying a more homogeneous nuclear distribution resulting in the mislocalization of speckle components and altered patterns of AS of pre-mRNAs [74,75,76]. *MALAT1/NEAT2* binds to the SRSF1 splice factor through its RRM domain [77,78]. A correct phosphorylation/dephosphorylation cycle of SR proteins is fundamental to ensure the proper nucleocytoplasmic transport of mRNA–protein complexes (mRNPs). When SRSF1 is phosphorylated, it accumulates in nuclear speckles; while its dephosphorylation favors the interaction with mRNAs, transport and accumulation in the cytoplasm [79,80]. Although the exact mechanisms through which *MALAT1/NEAT2*-interacting with SRSF1 modulates the phosphorylated/dephosphorylated ratio of SR proteins remains unclear, it might occur through interaction with PP1/2A phosphatases or with the SRPK1 splice factor kinase [81,82,83] or alternatively, by the direct interaction with *MALAT1/NEAT2* [73] (Figure 5a). Beyond AS, controlled levels of phosphorylated SR proteins are also likely to regulate other SR-dependent post-transcriptional regulatory events such as RNA export, nonsense mediated decay, and translation [77,81]. Interestingly, additional studies have also shown that *MALAT1/NEAT2* can hybridize with many nascent pre-mRNAs at active gene loci and participate in pre-mRNA splicing of such actively transcribed genes by recruiting SFs to the pre-mRNAs [84]. Furthermore, according to the psoralen analysis of RNA interactions and structures (PARIS) [85] and to the more recent developed RIC-seq application [86], multiple interaction sites exist between *MALAT1* and the spliceosomal RNA, U1snRNA, raising the possibility that *MALAT1/NEAT2* influences RNA processing through the recruitment or modification of other proteins localized to these sites.

*MALAT1/NEAT2* is abundantly expressed and widely associated with a variety of cancers. In hepatocellular carcinoma, *MALAT1/NEAT2* acts as a proto-oncogene through Wnt pathway activation and transcriptional induction of SRSF1. The latter leads to the over accumulation of its active form in the cell nucleus and the modulation of SRSF1 splicing targets, including the anti-apoptotic AS isoforms of S6K1 [87]. In colorectal cancer, instead, *MALAT1/NEAT2* triggers tumor growth and metastasis by binding to the splicing factor SFPQ causing the subsequent disruption of the splicing regulator complex SFPQ-PTBP2 and the release of the oncogene PTBP2 [88].

During adipocyte differentiation, the 4 kb lncRNA *NEAT1* tethers the SR protein SRp40 (now known as SRSF5) and retains it in paranuclear bodies to fine-tune the relative abundance of mRNA isoforms of the major transcription factor driving adipogenesis, *PPARγ*. It has been observed that the *NEAT1*-SRp40 association enhances SRp40 phosphorylation by CLK1 splicing factor kinase activity [89]. Conversely, depletion of *NEAT1* upon drug or siRNAs treatment, causes a decrease of both *PPARγ* isoforms (*PPARγ1* and especially *PPARγ2*) and SRp40 phosphorylation impairment, respectively. Furthermore, while SRp40 depletion resulted in deregulation of both *PPARγ* isoforms and, predominantly of *PPARγ2* mRNA levels, its overexpression increased exclusively *PPARγ2*. Therefore, fluxes in *NEAT1* levels during adipogenesis seem to modulate AS events likely by controlling the availability of phosphorylated SRp40 thereby affecting *PPARγ* splicing [90] (Figure 5b).

Another lncRNA abundantly localized to nuclear bodies is the lncRNA *Gomafu/RNCR2/MIAT* which is expressed in a distinct set of neurons in the mouse retina [91,92] and implicated in retinal cell specification [93,94] brain development [95] and post-mitotic neuronal function [92,96]. *Gomafu* was found to interact directly with the splicing factors QKI and SRSF1 and its dysregulation leads to aberrant AS patterns that resemble those observed in schizophrenia-associated genes (*DISC1* and *ERBB4*) [97]. In addition, *Gomafu* harbors a conserved tandem sequence of UACUAAC motifs that binds the splicing factor SF1, an early stage player of spliceosome assembly [98]. Furthermore, the splicing factor Clf3 was found to interact specifically with *Gomafu* in RNA–protein complexes containing the splicing factors SF1 and localize in specific nuclear bodies named CS bodies in the neuroblastoma cell line Neuro2A [99,100]. It has been proposed that *Gomafu* regulates splicing efficiency by changing the local concentration of SFs by sequestering them to separate regions of the nucleus [98] (Figure 5c).

An additional example of how lncRNAs may hijack SFs to fine-tune AS is the lncRNA *LINC01133.* This lncRNA binds the AS factor SRSF6, which induces EMT in colorectal cancer. By sequestering SRSF6 from other mRNA substrates, *LINC01133* modulates SRSF6 activity and reshapes the population of AS isoforms of SRSF6 mRNA targets which finally leads to the inhibition of EMT and metastasis [101] (Figure 5d). Similarly, the lncRNA *PNCTR,* over-expressed in a variety of cancer cells, contains hundreds of short tandem repeats to bind and sequester a consistent fraction of PTBP1 in the perinucleolar compartment [102]. This prevents PTBP1 from influencing splicing and therefore PTBP1-dependent pro-apoptotic events [103,104,105] (Figure 5e).

LncRNAs that act as sponge molecules can extensively rewire post-transcriptional gene regulatory networks by uncoupling the protein–RNA interaction landscape in a cell-type-specific manner. A recent study showed that the loss of 39 lncRNAs causes many thousands of skipped exons and retained intron splicing events affecting a total of 759 human genes at the post-transcriptional level. Interestingly, the alternatively spliced events were found associated with RBPs binding in proximal intron–exon junctions in a cell-type-specific manner [106]. Similarly, the natural antisense *TPM1-AS*, reverse-transcribed from the fourth intronic region of the tropomyosin I gene (*TPM1*), regulates *TPM1* alternative splicing through interaction with RNA-binding motif protein 4 (RBM4). The interaction prevents the binding of RBM4 to *TPM1* pre-mRNA and inhibits *TPM1* exon 2a inclusion (Figure 5f) [107]. Plant lncRNAs are also able to modulate AS by hijacking RBPs from their targets. In *A. thaliana*, an important number of intron retention events and a differential 5′ or 3′-end have been observed in a subset of genes in the plant-specific AS regulators (NSRa and NSRb) mutant compared to wild type plants [108]. In vitro experiments suggested that the lncRNA *ASCO* competes with other mRNA-target for its binding to these NSR regulators [109]. More recently, researchers analyzed the genome-wide effect of the knock-down and overexpression of *ASCO* and found a large number of deregulated and differentially spliced genes related to flagellin responses and biotic stress [110]. During this splicing process, *ASCO* interacts with multiple SFs including the highly conserved core spliceosome component PRP8a and another spliceosome component, SmD1b (Figure 5g). The NSR’s closest homolog in the model legume *Medicago truncatula*, MtRBP1/MtNSR1, has been characterized as a protein partner of the highly conserved and structured lncRNA *ENOD40*, which participates in root symbiotic nodule development [111]. *ENOD40* appears to re-localize MtRBP1 from nuclear speckles into cytoplasmic granules during nodulation thereby modulating MtRBP1-dependent splicing events [112] (Figure 5h).

SF-associated lncRNAs might also influence a specific splicing outcome depending on a given cellular context. For example, the prostate-specific lncRNA *PCGEM1* can mutually bind the splicing factors hnRNP A1 (silencer) and U2AF65 (enhancer) with opposite effects. While its interaction with hnRNP A1 suppresses the expression of androgen receptor (AR) splice variants such as AR3 by exon skipping, the interaction of *PCGEM1* with U2AF65 promotes AR3 by exonization and favors castration resistance [113]. In the brain, the cytoplasmic 200 long non-coding RNA *BC200* (*BCYRN1*) prevents apoptosis by modulating AS of a member of the Bcl-2 family proteins, the *BCL-x* gene [114]. AS of *BCL-x* leads to opposite effects on apoptosis when processed in either *BCL-xL* (anti-apoptotic) or *BCL-xS* (pro-apoptotic) [115]. Whereas *BC200* overexpression promotes *BCL-xL*, its depletion induces *BCL-xS* formation. A 17-nucleotide complementary sequence to *BCL-x* pre-mRNA in *BC200* appears to facilitate its binding to the pre-mRNA and promotes the recruitment of the hnRNP A2/B1 splicing factor. HnRNP A2/B1 binding interferes with association of *BCL-x* pre-mRNA with the *BCL-xS*-promoting factor Sam68 [116], leading to a blockade of Bcl-xS expression and anti-apoptotic conditions [117] (Figure 5i). Another example of a cellular context that causes isoform switching through lncRNAs is that of fibroblast growth factor receptors. FGF-2-sensitive cells arise following *lnc-Spry1* depletion. This lncRNA acts as an early mediator of TGF-β signaling-induced EMT and regulates the expression of TGF-β-regulated gene targets. However, *lnc-Spry1* has also been found to interact with the U2AF65 pyrimidine-tract binding splicing factor suggesting a dual role in affecting both transcriptional and post-transcriptional gene regulation in epithelial cells promoting a mesenchymal-like phenotype [118]. Recently, a link between stress-induced lncRNAs and AS has also been shown. The lncRNA *LASTR*, elevated in hypoxic breast cancer, is upregulated through the stress-induced JNK/c-JUN pathway. It interacts with SART3, a U4/U6 snRNP recycling factor, and promotes splicing efficiency. Depletion of *LASTR* leads to increased intron retention, with the resulting downregulation of essential genes to the detriment of cancer cells [119].

Ribosomal and RNA splicing complexes components, including YBX1, PCBP1, PCBP2, RPS6 and RPL7, have been shown to bind *LINC-HELLP*, a lncRNA implicated in the pregnancy-specific *HELLP* syndrome, through a splicing-mediated mechanism that is largely unknown. *HELLP* patient mutations within *LINC-HELLP*, alter the binding with these proteins depending to their location and negatively affect trophoblast differentiation. While mutations occurring from the 5′-end up to the middle of the *LINC-HELLP* are likely to cause loss of partner protein interactions, those at the far 3′-end increase their binding [120]. Among a cohort of breast cancer-associated and oestrogen-regulated lncRNAs, *DSCAM-AS1* has been recently found to be associated with tumor progression and tamoxifen resistance [121]. Researchers found over 2085 splicing events regulated by *DSCAM-AS1*, including alternative polyadenylation sites, 3′ UTR shortening and exon skipping events. *DSCAM-AS1* affects target gene expression and causes changes in the AS by interacting with hnRNPL which appears to mediate the exon skipping and 3′ UTR usage by a mechanism not yet fully elucidated [121].

**Figure 6:**
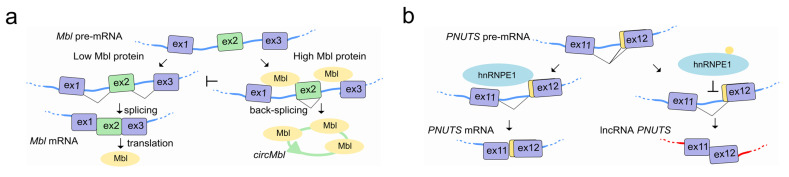
LncRNAs regulate pre-mRNA splicing by competing for splicing factors during their own splicing. (**a**) Left, In the presence of low amounts of *Mbl* (orange), the *Mbl* transcript is canonically spliced into a translatable mRNA encoding the Mbl protein. Right, when Mbl levels are high, Mbl binds to the pre-mRNA at the intronic regions flanking exon 2 and causes the exon2 back-splicing into *circMbl* (green), thereby preventing linear splicing and translation of the Mbl protein. *CircMbl* can also sequester Mbl protein, lowering its free cellular concentration, thereby providing a feedback mechanism to regulate Mbl levels. (**b**) The *PNUTS* gene can encode either the *PNUTS* mRNA or the lncRNA *PNUTS* depending on the usage of the 3′ alternative splice site located at the 5′-end of exon 12 which leads to the change of the ORF and the generation of a premature stop codon. Left, upon the binding of hnRNP E1 to a BAT consensus element located in the alternative splicing site that mask and prevents its usage, *PNUTS* pre-mRNA is spliced into *PNUTS* mRNA then translated into the PNUTS protein. Right, loss of hnRNP E1 binding to the alternative splice site uncovers the consensus element and allows its usage by the spliceosome machinery to achieve the splicing to yield the lncRNA *PNUTS* transcript.

Canonical splicing of the linear pre-mRNA can compete for SFs with circularization of exons in circRNAs by mechanisms that are tissue specific and conserved in animals [122]. In flies and humans, the second exon of the SF muscleblind (Mbl (fly)/MBNL1 (human)) is circularized in *circMbl*. The introns flanking this circRNA as well as the circRNA itself contain highly conserved Mbl/MBNL1 binding sites, which are strongly and specifically bound by Mbl. Modulation of Mbl levels regulates the splicing of its own pre-mRNA into *circMbl*, and this in turn relies on Mbl binding sites [123] (Figure 6a). A circRNA proposed to act as an angiogenesis regulator by sponging SFs, is *circSMARCA5*. *CircSMARCA5* interacts with SRSF1 and promotes the switching from pro- to anti-angiogenic splicing isoforms of VEGF-A in glioblastoma multiforme, representing an opportunity to develop a novel anti-angiogenic cancer therapy [124]. Interestingly, circRNAs have been also found associated with the splicing factor QKI during human EMT [123], and correlate with exon skipping throughout the genome in human endothelial cells [125].

LncRNAs can also interact with SFs to regulate their own splicing as is the case with the lncRNA *PNUTS,* also known as a competitive endogenous RNA (ce-RNA). The *PNUTS* gene can express a regular *PNUTS* mRNA encoding for the protein phosphatase 1 binding protein, but also to an alternatively spliced non-coding isoform called lncRNA-*PNUTS* with a distinct biological function. While *PNUTS* mRNA is ubiquitously expressed, the lncRNA-*PNUTS* one is more tumor-relevant and generally serves as a competitive sponge for miR-205 during EMT. The splicing decision to produce either mRNA or lncRNA relies on the binding of hnRNP E1 to a structural element located in exon 12 of *PNUTS* pre-RNA. Once released from this structural element, hnRNP E1 translocates from the nucleus to cytoplasm, allowing the AS and generation of the non-coding isoform of *PNUTS* to take place [126] (Figure 6b).

## 6. Concluding Remarks and Future Perspectives

Growing evidence suggests that lncRNAs control the regulation of AS in response to several physiological stimuli or during disease processes through changes in chromatin conformation, or by interfering with the overlapping antisense genes, genomic loci or SF activity. LncRNA antisense transcription pausing and elongation, as well as the capability of sponging RBPs, can also result in altered mRNA splice isoform expression patterns. The recent discovery of the circRNAs has also shown how a special class of lncRNAs can wholly integrate with the splicing process itself, affecting the splicing outcome of their linear cognates.

Some aspects of lncRNA-mediated AS regulation remain mostly unexplored. For instance most lncRNA sequences are not conserved across species, suggesting that most of their functionality might relies on their RNA structure. The role played by lncRNA secondary structure in determining their ability to regulate AS remains poorly investigated. Moreover, mRNA methylation is known to impact on AS by affecting the accessibility of hnRNPs to pre-mRNAs. Specifically, N6-methyladenosine (m6A) can serve as a switch to regulate gene expression and RNA maturation [127]. The existence of an interplay between RNA methylation and long non-coding RNA also raises the question of whether or not lncRNAs play a role in recruiting or reading mRNA methylation during AS processes. Furthermore, m6A modifications that occur on lncRNAs and circRNAs might change their function in AS regulation by providing a binding site for the m6A reader proteins or by modulating their structure–all of these questions remain unanswered.

Over the past years, our understanding of the mechanisms through which lncRNAs affect gene expression has been limited by their intrinsic properties (mainly length and low expression) and the lack of powerful experimental assays. With the increasing prevalence of splicing events and the discovery of over a hundred thousand lncRNAs, it is likely that the involvement of lncRNAs in regulating AS is far greater than the currently known. Further research is needed to gain a deeper understanding of how lncRNAs contribute to the regulation of AS in development and disease.

**Table 1:** List of lncRNAs involved in splicing regulation.

LncRNA Name	Splicing Target	Splicing Mechanism	Regulatory Effect or Associated Disease	Ref
LncRNAs regulating AS by chromatin modifications				
*asFGFR2*	*FGFR2*	Recruiting Polycomb complexes and KDM2a to modify histone methylation and favor exon IIIb inclusion	Epithelial development	[23]
Antisense transcripts at each *Pcdh*α first exon	*Pcdh*α	First exon selection by histone modifications and distant DNA loop	Neuronal self-identity	[31]
LncRNAs regulate AS through DNA-RNA interactions				
*SEP3* exon6 circRNA (plant)	*SEP3*	Exon skipping through R-loop formation at exon 6	Flowering time	[44]
LncRNAs regulate AS through RNA-RNA interactions				
*NCYM* NAT	*NMYC*	Intron I retention via antisense-sense RNA-RNA duplex	Cancer	[57]
*NR1D1*	*THRA*	Favoring α1 isoform by forming antisense-sense RNA-RNA duplex with the α2 mRNA	Thyroid hormone-responsiveness	[58,59]
*SAF*	*FAS*	Exon 6 skipping by forming RNA-RNA duplex with the target pre-mRNA and recruiting SPF45	Cancer Apoptosis	[60]
*ZEB2* NAT	*ZEB2*	Preventing splicing of the IRES-containing intron through RNA-RNA interaction with the mRNA	EMT	[61]
*51A*	*SORL1*	Splicing shift from A to variant B by antisense-sense RNA-RNA duplex with an intronic sequence of the pre-mRNA	Alzheimer	[67]
*17A*	*GPR51*	Splicing shift from full-length to shorter GABAB R2 variant by antisense-sense RNA-RNA duplex	Alzheimer	[68]
LncRNAs regulate AS by modulating the activity of splicing factors				
*MALAT1/NEAT2*		Modulation of SR localization and phosphorylation Uncoupling PTBP2 from SFPQ-PTBP2	Cancer	[73,88]
*NEAT1*	*PPARγ*	By interacting with CLK1 kinase to modulate SRp40 phosphorylation status	Adipocyte differentiation	[71,89,90]
*Gomafu/RNCR2/* *MIAT*		Interaction with QKI and SRSF1 Association with SF1 Localization of SF1 and Clf3 in CS bodies	Schizophrenia Retinal cell and brain development Post-mitotic neuronal function	[97,98,99,100]
*LINC01133*		Interaction and titration of SRSF6 splicing factor from target genes	EMT	[101]
*PNCTR*		Hijacking PTBP1 in the perinucleolar compartment	Cell survival	[102]
*TPM1-AS*	*TPM1*	Splicing shift to V1 or V3 isoforms by sequestering RBM4	Cancer	[107]
*ASCO* (plant)		Association with SmD1b and PRP8a and hijacking NSRa/b from the spliceosome	Lateral root formation	[109,110]
*ENOD40* (plant)		Control nucleocytoplasmic of MtRBP1	Symbiotic nodule development	[111,112]
*PCGEM1*		Mutual bond with either hnRNP A1 or U2AF65 to promote or suppress specific AR splice variants	Castration resistance	[113]
*BC200*	*BCL-x*	Interaction with pre-mRNA and recruitment of the hnRNP A2/B1 which prevent Sam68 association	Apoptosis	[115,117]
*Lnc-Spry1*		Interaction with U2AF65	EMT	[118]
*LASTR*		Promoting splicing efficiency by interacting with SART3	Stress-induced JNK/c-JUN pathway	[119]
*LINC-HELLP*		Interaction with ribosomal and splicing complex components (eg: YBX1, PCBP1, PCBP2, RPS6 and RPL7)	*HELLP* syndrome	[120]
*DSCAM-AS1*		Exon skipping and 3′ UTR usage by interaction with hnRNPL	Tumor progression and anti-estrogen resistance	[121]
*CircMbl*	*Mbl*	Competing with the linear cognate by sequestering Mbl protein	Neuron Development	[123]
*CircSMARCA5*		Interaction with SRSF1 and promotion of the anti-angiogenic splicing isoforms of VEGF-A	Angiogenesis	[124]
*PNUTS*	*PNUTS*	Self-splicing regulation modulating the activity of hnRNP E1	EMT	[126]

## Abbreviations

Alternative splicing (AS); splicing factor (SF); RNA binding proteins (RBPs); long non-coding RNAs (lncRNAs); RNA polymerase II (RNAPII); CCCTC-binding factor (CTCF); Natural Antisense Transcripts (NATs); epithelial-mesenchymal transition (EMT); Alzheimer’s disease (AD); amyloid precursor protein (APP); circular RNAs (circRNAs); N6-methyladenosine (m6A).
